# The Integration of Primary Care and Public Health in Medical Students’ Training Based on Social Accountability and Community-Engaged Medical Education

**DOI:** 10.3389/ijph.2023.1605359

**Published:** 2023-01-26

**Authors:** Jaime Kristoffer Punzalan, Monserrat Guingona, Mary Germeyn Punzalan, Fortunato Cristobal, Annika Frahsa, Harvy Joy Liwanag

**Affiliations:** ^1^ Ateneo de Zamboanga University School of Medicine, Zamboanga City, Philippines; ^2^ Institute of Social and Preventive Medicine, University of Bern, Bern, Switzerland

**Keywords:** primary care, public health, medical education, social accountability, community health, health systems

## Abstract

**Objectives:** Primary care and public health comprise the bedrock of health systems, but their divergence has produced two groups of practitioners either focused on individual health or population health. We explored how primary care and public health were integrated in medical students’ training in Zamboanga Peninsula, Philippines.

**Methods:** Our qualitative study reviewed community health plans in two municipalities and thematically analyzed the perspectives of medical students, faculty, alumni, and community stakeholders through focus group discussions and in-depth interviews.

**Results:** Integration began by operationalizing a curriculum requiring medical students to serve rural communities during most of their training—a departure from the conventional, hospital-based medical education in the Philippines. The medical students’ community immersion provided opportunities for integrating primary care and public health activities that influenced their personal orientations and the health situation in communities. Integration continued after training as alumni found themselves serving as primary care and public health practitioners in the region.

**Conclusion:** Social accountability and community-engaged medical education provided the foundation for medical students to integrate primary care and public health in practice to respond to local needs.

## Introduction

Medicine and public health diverged due in part to the separate development of medical and public health schools in the United States in the early 1900s [1]. Public health evolved as the art and science of preventing disease, prolonging life, and promoting population health and wellbeing [2]. The goals of medicine are similar—except for its focus on individual health [3] with primary care, in particular, concerned with provision of services addressing personal healthcare needs through patient partnership within the context of family and community [4]. From training to professional regulation, medicine and primary care on the one hand, and public health on the other hand, developed their respective curricula, core competencies, and scopes of practice. In this article, we consider primary care [5] an essential element of the practice of medicine and focus on primary care when discussing the divergence between medicine and public health.

Conventional medical curriculum in the Philippines leading to a Doctor of Medicine (MD) degree is a 5-year program dominated by a focus on basic and clinical disciplines, such as human anatomy, pathology, internal medicine, pediatrics, obstetrics, and general surgery, with little coverage for community practice and public health [6]. Typically, the final year is dedicated to clinical training in hospital settings with at most 2 months dedicated to service in communities. The conventional medical education system has inadvertently influenced more physicians to serve in hospitals in urban settings, resulting in an inequitable health workforce distribution that adversely affects rural areas with less health workers [7]. More physicians also choose to train to become specialists rather than practice as primary care providers, which has led to calls to reorient health professionals’ education in the Philippines towards Primary Healthcare [8] as emphasized in the country’s Universal Healthcare (UHC) law [9]. The Philippines has also lost a significant number of health professionals through migration to high-income countries where there are better working conditions and higher salaries [10]. We argue that healing the divergence of primary care and public health by integrating these functions in practice will contribute to addressing these challenges.

The Zamboanga Peninsula is a region in the Philippines with severe socioeconomic and health situations. In 1994, poverty incidence was 50.6% of the population [11], infant mortality rate was 15.1 per 1,000 live births [12], and non-skilled attendants delivered 54.1% of births [12]. Established in the same year, the Ateneo de Zamboanga University School of Medicine (ADZU-SOM) is a private, non-profit medical school with the primary mission to train medical students who will respond to the health needs of the region and contribute to the improvement of the overall health situation [13]. The ADZU-SOM medical curriculum is motivated by *social accountability,* defined by the World Health Organization as “the obligation of medical schools to direct their education, research, and service activities towards addressing the priority health concerns of the community, region, and/or nation they have a mandate to serve” [14]. Furthermore, ADZU-SOM pioneered a model of community-engaged medical education (CEME) that emphasized interdependent and reciprocally beneficial partnerships between medical schools and the communities they serve—a step farther from models of community-oriented and community-based medical education [15]. Most medical students recruited from the region were financially supported by philanthropies to keep tuition and fees affordable [16].

The ADZU-SOM medical curriculum differs from the conventional medical curriculum since medical students devote significantly more time immersing in the region’s rural communities during the entirety of their training [17]. Medical students typically serve their assigned communities for 2 months in the first year, 2 months in the second year, 1 month in the third year, and 10 months in the fourth year. During these community immersions, students reside in the communities and collaborate with community stakeholders to identify and address health priorities. Once identified, students and communities devise acceptable, culturally appropriate interventions to improve the community health situation. The fourth year includes the monitoring and evaluation of community health plans (CHP) that began in the first year with an aim to improve the community health situation. In addition to CHP, medical students also conduct individual research projects on solutions to specific community health problems. Graduates earn a complementary Master of Public Health (MPH) degree after an additional fifth year when they complete a thesis project that draws on the public health coursework integrated throughout the 4 years of the MD program [18].

From only 15 physicians in the first batch of graduates in 1999, about 35 physicians graduate from ADZU-SOM per year with over 80% of alumni practicing in Zamboanga Peninsula [16, 19]. When compared with graduates of the conventional medical curriculum, a study has shown that ADZU-SOM graduates were more likely to work as government physicians than in private health facilities and as generalists than specialists [20]. Although lessons from the ADZU-SOM experience were previously described in the literature [15–28], none has explored how primary care and public health could be integrated during medical students’ training based on social accountability and CEME. Since there are calls to integrate primary care and public health for optimal health systems [29], more evidence is needed about how the functions could come together in practice to achieve potential synergies for enhancing individual- and population-level health [30]. Many of the models for integrating primary care and public health that have been described in the literature draw on experience from the United States and Europe [31] including: public health professionals embedded in primary care teams; public health practitioners and primary care providers working together; inclusion of preventive services in the health service benefits of individual patients; provision of primary care services within public health settings; incentivizing prevention within primary care services; and multi-disciplinary training of primary care practitioners who undertake additional public health training [32]. However, the role of training as a foundation where competencies in primary care and public health are simultaneously developed in the same group of practitioners who can perform both functions in health systems remains unexplored.

We reviewed CHP in two municipalities and explored the perspectives of medical students, faculty, and alumni, as well as community stakeholders and analyzed how primary care and public health were integrated during medical students’ training based on social accountability and CEME.

## Methods

### Study Design and Participants

**FIGURE 1:**
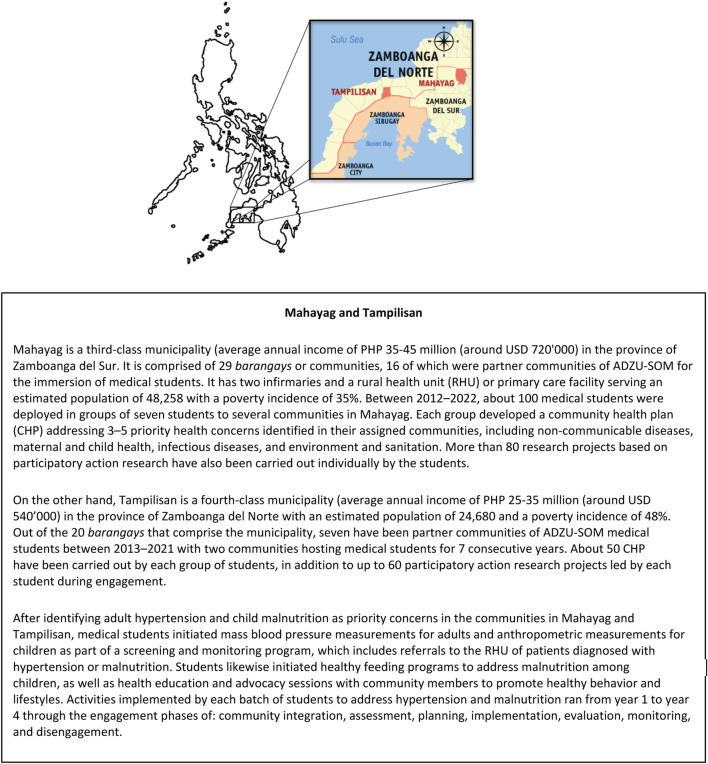
Map of the municipalities of Tampilisan and Mahayag in the southern Philippines; adapted from map by Mike Gonzalez (Zamboanga Peninsula, Philippines. 2022).

This was a qualitative study set in the Zamboanga Peninsula region in the Philippines where ADZU-SOM medical students have served rural communities since the school was established in 1994 (Figure 1). We purposively selected two rural municipalities in the region—Mahayag and Tampilisan—because from 2009 to 2021, they cumulatively hosted 100 and 60 medical students, respectively—which were the highest numbers for any partner municipalities of the school.

For focus group discussions (FGD) and in-depth interviews (IDI), we sought participants from four different groups of key informants: (a) medical students; (b) faculty members and faculty community preceptors; (c) community stakeholders; and (d) program graduates or alumni. We randomly selected medical students from a list of students who served in communities in Mahayag and Tampilisan between 2019 and 2021. For faculty members and community preceptors, selection was purposive to prioritize those employed at ADZU-SOM for 5–25 years and directly involved in curricular content and community engagement, planning, and oversight. We used convenience sampling in selecting community stakeholders and program graduates or alumni. Community stakeholders in the FGD previously worked directly with medical students in various implemented activities who were available during our field visits. We approached study participants by phone or in-person. In total, 44 key informants participated in our study. Supplementary File S1 summarizes study participant demographic characteristics.

### Community Health Plan Review and Context

For the initial study phase, we reviewed 24 CHP for Mahayag and Tampilisan. CHP topics included activities for addressing hypertension and malnutrition—health issues that medical students prioritized during their community immersion in consultation with stakeholders. We used an activity checklist (Supplementary File S2) developed by the team to extract information about: a) activities implemented by medical students in communities; b) primary care or public health category of activity; c) population target; d) how activities were carried out; e) outcomes achieved; and f) challenges encountered. We also referred to the grey literature for the overall health situation in Mahayag and Tampilisan. We arranged and visualized medical student activities in a timeline illustrating primary care and public health activities and briefly described the socioeconomic and health situation in narrative form.

### Focus Group Discussion and In-Depth Interview Guides

We developed FGD and IDI guides (Supplementary File S3) that were pilot tested during the first FGD. The FGD and IDI guides were designed to allow exploring perspectives on integration more broadly beyond the issues of hypertension and malnutrition. Sample questions for the FGDs and IDIs included:

(a) Medical students
  • What is your understanding of primary care and public health, including their similarities and differences?
  • Can you recount a particular experience (during community engagement) when you applied or integrated primary care and public health?
(b) Faculty members or faculty community preceptors
  • Can you describe how primary care and public health concepts/activities are integrated in the curriculum?
  • What influence do you think did the integration of primary care and public health have on students’ commitment to address community health needs?
(c) Community stakeholders
  • Can you describe benefits from student-led primary care and/or public health activities?
  • What are the key changes you have seen in the community since the students’ service in your community?
(d) Graduates/alumni
  • Can you recall an experience when you integrated primary care and public health as medical students and what outcomes or impact did it result in?
  • Can you describe how the experience of integrating primary care and public health prepared you to perform your functions in your current role in the health system?

### Conduct of FGD and IDI

In total, we conducted five FGD sessions with 40 participants. Two FGD sessions were with 13 medical students, one FGD session was with six faculty members and community preceptors, and two FGD sessions were with 21 community stakeholders. We conducted four IDI sessions with two faculty members and two program graduates/alumni. Most FGD participants were female (24/40), while IDI participation was evenly split (two females and two males). We determined data saturation [33], or when new insights were no longer obtained from the conversations, after five FGDs and four IDIs.

Three co-authors (JP, male, MD, MPH; MG, female, MD, MPH; MP, female, MD, MPH) with experience in qualitative research conducted most of the FGD and IDI either in-person or virtually through Zoom (Zoom, San Jose CA, United States) using a mix of English and the local dialect, whenever appropriate (note: English is widely spoken in the Philippines). During data collection, these co-authors were affiliated with ADZU-SOM as faculty members and knew some of the study participants. We performed the FGD with community stakeholders in the local dialect and exclusively in-person during field visits to the communities since most community members did not have computers or internet connections for a virtual interaction.

### Data Analysis

We based our analytical process on Gale’s Framework Method [34]. We audio recorded and transcribed the FGD and IDI in Microsoft Word using Trint^TM^ (Trint, London, United Kingdom). MP—fluent in the local dialect—translated community stakeholder transcripts from the local dialect into English. All final transcripts were in English. We coded text from the transcripts on Microsoft Word. We performed subsequent synthesis into categories and themes on Microsoft Excel (Microsoft, Redmond WA, United States).

We inductively approached thematic analysis by analyzing individual perspectives from study participants, identifying patterns in responses, and grouping their ideas into broader categories. We identified themes with a focus on integration mechanisms for primary care and public health in community settings, including facilitating factors and barriers to integration, while including findings from CHP review. Three co-authors (JP, MG, MP) performed the coding and thematic analysis. We recognized potential bias during data collection and analysis from the role of interviewers and some of the study participants as faculty members or students at the school. Three virtual meetings for reflexivity sessions [35] with other collaborators (AF, female, Dipl-Pol, PhD; HL, male, MD, MBA, PhD) who were external to the school provided space for constructive exchanges on identified themes and for challenging potential biases in data interpretation. Illustrative quotes from the transcripts were selected to highlight study themes. Study participants did not provide feedback to findings. We report our methods and findings according to COREQ guidelines [36].

### Ethical Considerations

The Ateneo de Zamboanga University Research Ethics Committee (Protocol Code: 2022-0077) approved our study protocol. Prior to FGD and IDI, we secured written informed consent from participants. To ensure study participant anonymity, we replaced personal identifiers with numbers during transcription. We stored audio files, transcripts, and coding files in Google Drive (Google LLC, Mountain View CA, United States) with access restricted to three co-authors (JP, MG, HL). Files would be maintained in cloud storage for a maximum of 5 years in compliance with the Data Privacy Act of the Philippines [37].

## Results

**FIGURE 2:**
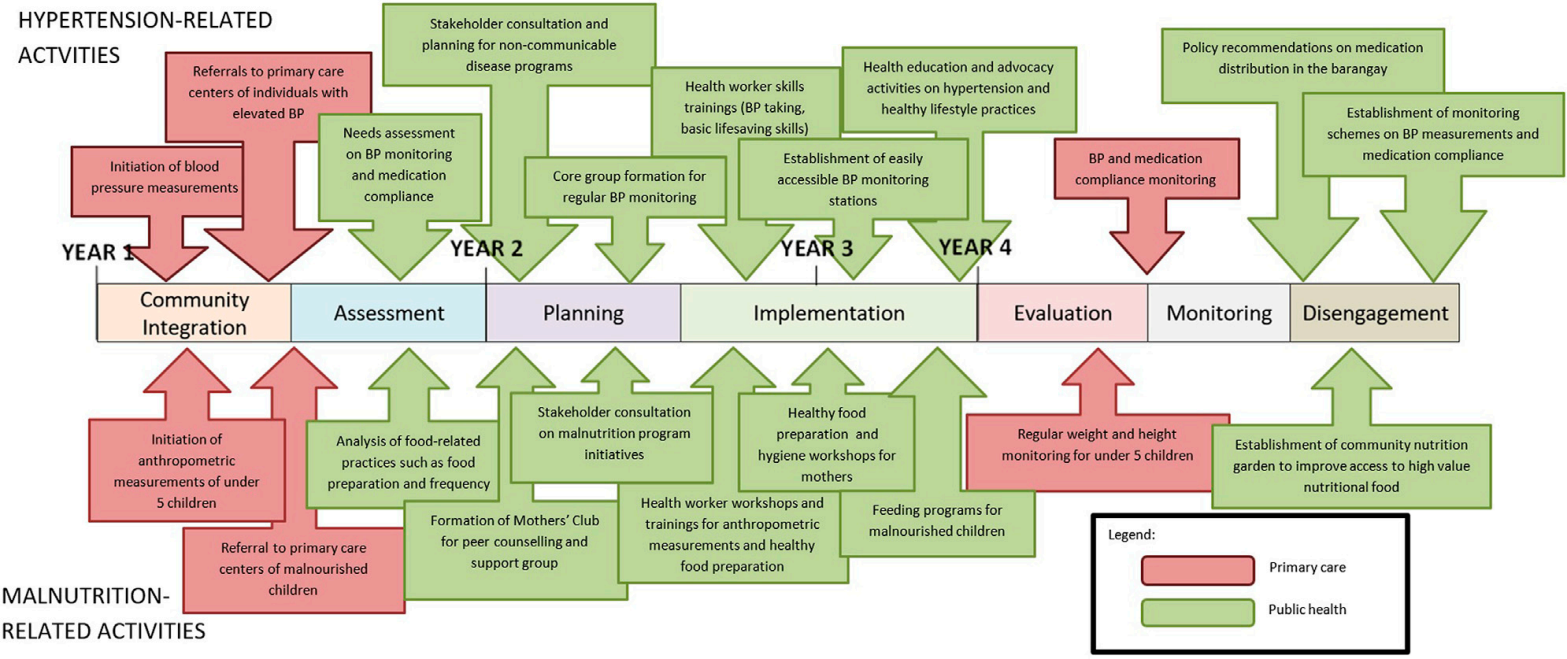
Overview of primary care and public health activities carried out by the medical students in the communities of Mahayag and Tampilisan (Zamboanga Peninsula, Philippines. 2022).

Our review of CHP for communities in Mahayag and Tampilisan revealed that medical students performed a combination of primary care and public health activities to address hypertension and malnutrition throughout their community engagement (Figure 2). Primary care activities in the community targeted individual health and included visits to households for monitoring blood pressure, weight, and height, as well as referrals of patients with hypertension and malnutrition for further management in rural health units or district hospitals. Public health activities targeted population health, including capacity building, resource allocation, and health education for behavior change.

Our analysis of study participants’ perspectives revealed three main themes for the integration of primary care and public health: a) curriculum as the source of integration; b) factors enabling integration; and c) outcomes of integration.

### Curriculum as the Source of Integration

Among study participants, there was consensus that social accountability and CEME drove teaching and learning activities that enabled the integration of primary care and public health since the curriculum deliberately brought the functions together. Integration in practice was the result of operationalizing the curriculum in the communities. Instead of being discipline-based, the curriculum was outcome-based that ensured graduates acquired competencies that enabled them to practice primary care and public health effectively in areas with greatest need.

The needs of the community and the broader health system became the basis for determining requisite competencies that students must acquire during training (Table 1). This view was echoed by a faculty community preceptor who said: “The integration starts in the classroom during the first year. The medical students are taught the competencies of a physician that allow them to integrate primary care and public health” (note: illustrative quotes were edited for brevity, clarity, and correctness). The same view was shared by a medical student who said that “The curriculum allows us [medical students] to understand the roles of a physician that is not limited only to being clinicians.” A faculty member remarked that “Public health concepts are introduced early in the academic year, then clinical aspects are introduced the next year.” During the first semester of the first year, two-thirds of content taught to students comprised population health concepts integrated with biomedical concepts.

**TABLE 1:** Selected quotes from participants about curricular components where public health and primary care are integrated (Zamboanga Peninsula, Philippines. 2022).

Curricular component	Sub-category	Codes (frequency of statement) and example participant quotes^a^
Curricular goals	Competency-based medical education	Competencies enabling integration of PC and PH (3)
		“Integration starts in classroom. During freshman year, medical students are taught physician competencies that allow them to integrate PC and PH.”—Community preceptor 1
		“We teach the basic roles of a physician-clinician, teacher-learner, manager, researcher.”–Faculty 2
		“The curriculum allows us to understand the roles of a physician that is not only limited to a clinician.”—Medical student 3
	Research requirements	Research focus (2)
		“Our [medical student] research integrates PC and PH because they aim to solve community health problems.”—Medical student 1
Teaching-learning methods	Community health management course	Community engagement (21)
		“In communities, [medical students] integrate PC and PH. During the first community visit, they conduct blood pressure monitoring which by nature is PC. They then collate the data during the blood pressure monitoring, analyze the data, and interpret it. I consider this PH. They then come up with activities that are PC and PH.”—Community preceptor 2
		“Community engagement offers avenues where competencies enabling PC-PH integration are honed, like communication skills.”—Faculty 4
		“PC is integrated with PH since students are providing PC services through district hospital rotations and by manning local health stations.”—Faculty 5
		“Student engagement in the community does not only take place in the *barangay* [community]. Students are also encouraged and required to rotate within district hospitals responsible for delivering PC to rural communities.”—Community preceptor 1
	Internship/clerkship	Hospital rotations (3)
		“We also integrate PH in hospital rotations. We conduct health teaching to patients in the wards.” —Medical student 3
Instructional material	PBL modules	Population health perspective in PBL (2)
		“PBL modules incorporate population health perspectives.”—Faculty 2
	Content	Content (4)
		“PH concepts are introduced early in the academic year, then clinical aspects are introduced the next year.” —Faculty 4

^a^ Edited for brevity, clarity, and correctness.

PBL, problem-based learning; PC, primary care; PH, public health.

Most study participants agreed that primary care and public health were integrated through the community immersions as designed by the curriculum. Medical students spend 10 months of the fourth year of the program immersed in their assigned communities. During community immersions, medical students actively collaborated with local stakeholders (formal and informal community leaders) to assess health needs at individual levels and plan solutions to address these needs at the level of population health. Plans later transformed into student research projects that assessed the impact of the proposed interventions in addressing the challenges identified. Students and communities implemented solutions together through combinations of primary care and public health activities (Table 2).

**TABLE 2:** Selected quotes from participants about student-driven activities during the community immersions (Zamboanga Peninsula, Philippines. 2022).

Health issue	Function	Activity	Community stakeholder participant quotes^a^
Non-communicable disease	Public health	Health teaching	“To help address malnutrition, students taught and encouraged households—especiallythose with children with malnutrition—to do backyard gardening.”—Community stakeholder 5
			“Their [medical student] activities, such as *Zumba* dance classes, for individuals with hypertension also targeted lifestyle modifications and low-fat low-salt diet.”—Community stakeholder 2
			“We also educated them [parents of children with malnutrition] on how they can effectively nourish their children because we were trained by the medical students.”—Community stakeholder 3
		Mass community screening	“Students went house-to-house taking blood pressure to find people with hypertension in the community.”—Community stakeholder 12
			“With the students, we went house-to-house weighing and monitoring children with malnutrition.”—Community stakeholder 3
		Infrastructure development	“The *barangay* health station was renovated with the help of the medical students.”—Community stakeholder 10
		Capacity building	“The *barangay* health workers were provided with a BP [blood pressure] apparatus and trained on how to conduct BP monitoring.”—Community stakeholder 12
	Primary care	Provision of medicines for at-risk individuals	“They [medical students] initiated programs for individuals with hypertension in the community. They referred them to the rural health unit for provision of antihypertensive medications.”—Community stakeholder 2
		Referral systems	“Students also listed names of individuals with hypertension and referred them to the rural health unit for further management.”—Community stakeholder 2
		Feeding program and deworming	“The students along with mothers conducted feeding programs for children with malnutrition.”–Community stakeholder 3
Communicable disease	Public health	Education-information campaigns	“We [community members] were also taught and trained on water purification methods to prevent potential diarrheal outbreaks.”—Community stakeholder 2
			“We [*barangay* officials and medical students] conducted responsible pet ownership training where we tagged and vaccinated dogs with anti-rabies vaccine. This was made possible through our coordination with the local department of agriculture.”—Community stakeholder 4
		Mass vaccination	“The students [medical] together with the *barangay* health workers and conducted mass flu vaccination for older residents.”—Community stakeholder 11
	Primary care	Referral systems	“Students created referral systems linking *barangay* to the local district hospital.”—Community stakeholder 3
Environmental health	Public health	Health teaching	“The students taught the residents about household waste segregation.”—Community stakeholder 1
			“Residents were also taught about the importance of an acceptable toilet facility.”—Community stakeholder 19
		Infrastructure development	“[Medical students] provided and improved the collection system and material recovery facility continually used today.”—Community stakeholder 8
		Capacity building	“They [medical students] helped us create our *barangay* disaster risk and management plan.”—Community stakeholder 8
			“Students organized basic life support training.”—Community stakeholder 9

^a^ Edited for brevity, clarity, and correctness.

A faculty community preceptor commented that primary care and public health were integrated in the process of devising solutions: “In the community, the [medical students] integrate both public health and primary care. During the first community visit, they conduct blood pressure monitoring which by nature is primary care. They then collate the data during blood pressure monitoring, analyze the data, and interpret the data to implement community interventions. This I consider as public health. They then come up with activities that are both primary care and public health.” Another faculty community preceptor shared that “Students are also encouraged and required to rotate within district hospitals responsible for delivering primary care to rural communities.” This was also required by the curriculum for the students to learn competencies that support them to function effectively in different practice environments.

### Factors Enabling Integration

Participants identified several systemic, organizational, and interactional factors enabling integration of primary care and public health during medical students’ community engagement (Table 3). Systemic factors refer to environments outside of organizations where integration happens, such as government support for school initiatives and an enabling policy environment ensuring that interventions match local health needs. As a faculty community preceptor put it: “During the community engagements, the students involved intersectoral partnerships within and among institutions in addressing local health needs. For example, they invited expert resource speakers to conduct health education in the community and partnered with [non-government organizations] for funding to support the activity.” Organizational factors refer to conditions within organizations [31], such as strong social accountability, highly skilled staff, and substantial community engagement duration that allow meaningful impact to take place*.* A faculty member commented: “The social accountability principles embedded in the course contents, teaching learning activities, and student requirements allow them [medical students] to integrate public health and primary care.” Finally, interactional factors refer to interfaces between stakeholders [38]. For successful integration, partners must share aspirations and purpose and recognize the importance of integrating primary care and public health. This was captured by the statement of a community stakeholder: “We agreed to be partners with the medical school and allowed students to engage with our community because of the purpose of the student engagements. We believe that they are here to help us address our health-related needs and we are here to help them in their learning.”

**TABLE 3:** Selected quotes from participants on enabling factors for integrating primary care and public health (Zamboanga Peninsula, Philippines. 2022).

Enabling factors	Sub-category	Codes (frequency of statement) and example participant quotes^a^
Systemic	Collaboration between communities and institutions	Mutual collaboration (4)
		“During community engagement, students involved intersectoral partners within and among institutions addressing health concerns, such as inviting experts as resource speakers to conduct health teachings in the community and partnering with NGOs for funding.”—Community preceptor 1
		“Community residents receptive to the partnership were a key to the success of the outcomes.”—Community preceptor 2
		“The success of student community engagement also relies on active participation from local leaders. If a local leader is supportive of the activities, then it will be successful.”—Community stakeholder 8
Organizational	Socially accountable medical education mandate	Social accountability (3)
		“The social accountability principles embedded in the course content, teaching-learning activities, and student requirements allow integrating PC and PH.”—Faculty 5
		“[Problem-based learning] modules, especially the Physician Manager module that early on describes the various physician roles, with a perspective that [being a] clinician is not the only role for physicians, is an enabling factor for integrating PC and PH.”—Alumnus 1
	Length of community engagement	Length of exposure (3)
		“More than 50% of the students’ academic year is spent in communities—ample time for meaningful integrations.”—Faculty 1
	Faculty member program graduates	Faculty commitment (2)
		“ADZU-SOM’s committed alumni faculty serve as role models for future career path choices.”—Faculty 5
Interactional	Shared purpose and beliefs between the community and institution	Shared purpose (4)
		“Rapport with the community is established at the start and set mutual goals.”—Medical student 11
		“We agreed to be partners with the medical school and allowed students to engage with our community because of the purpose of the student engagements. We believe that they are here to help us address our health and health-related needs, and we are here to help them in their learning.”—Community stakeholder 9

^a^ Edited for brevity, clarity, and correctness.

NGOs, non-government organizations; PC, primary care; PH, public health.

### Outcomes of Integration

Integrating primary care and public health in medical education influenced several outcomes at the level of individuals, communities, medical students, and the healthcare system (Table 4). The outcomes were attested by the medical students, faculty, and community stakeholders who shared a wide range of individual and community outcomes, including changes in health-seeking behaviors among community members and empowerment of community leaders. Outcomes for medical students included enriched academic experiences and the formation of values that enhanced their understanding of primary care and public health concepts, preparing them for future roles in the health system. As an alumnus shared: “The community immersions definitely prepared me for my professional role as an MHO (municipal health officer) and now working for the Department (Ministry) of Health. It is during the community immersions where I saw and experienced the healthcare system.”

**TABLE 4:** Selected quotes from participants on the outcomes of integrating primary care and public health (Zamboanga Peninsula, Philippines. 2022).

Main category	Sub-category	Codes (frequency of statement) and example participant quotes^a^
Individual/community outcomes	Changes in health-seeking behaviors	Change in behavior (4)
		“There is a change in community members’ behavior, practices, and knowledge.”—Community preceptor 1
	Empowered *barangay* leaders	Empowered leaders (4)
		“This shows the positive effects brought about by student activities and evidenced by the [local government unit] adopting some student-initiated programs and implementing them municipality-wide. Examples include the extension of the committee on health, the creation of community water wells to improve the water supply, and the disaster risk response program for flood control.”—Community preceptor 1
		“During the pandemic, students were unable to come back. I noticed this time *barangay* officials came and lobbied for health and health-related services for their *barangay*. For example, in *barangay* Camul the community leaders themselves lobbied for the renovation of their local health station.”—Community preceptor 2
		“The *barangay’s* active involvement in the process allowed students to guide the *barangay* on the process on how they can achieve better health and health-related outcomes. For example, involving them in data gathering and showing the community members the importance of data driven solutions, in looking for readily available resources, and in approaching stakeholders who can help. I have noticed that the community leaders now know how to attain better access to health-related resources. Community leaders before were more passive; now they know the process—they need to make policies and approach agencies, then they need to constantly follow-up. Now 70% of our *barangay* allocate budget for antihypertensive medications.”—Community preceptor 2
	Better health outcomes	Better health outcomes (4)
		“Individuals with hypertension had better control of their blood pressure. This was achieved because we brought services to their communities. We conducted health teaching to improve their health-seeking behavior. We referred them to the PC center if they needed to be further evaluated and managed.”—Medical student 3
Medical student outcomes	Appreciation of importance of PC and PH and their integration	PC and PH importance (9)
		“Students are encouraged to partner with the regional Department of Health to help improve delivery of local health services.”—Community preceptor 1
		“Graduates appreciate and better understand the realities in the community, appreciate the role of social determinants of health as factors.”—Faculty 3
		“The graduates have a better appreciation of the health system.”—Faculty 4
		“Community engagements allowed us to experience the health system.”—Alumnus 1
		“Medical students during the community engagements are viewed as PC service providers, and it is there where the students realize the importance of integrating PC and PH.”—Faculty 1
		“The community engagements showed us the realities of the problems that exist. We experienced the true root causes of the health needs, and this made us realize that these problems should be targeted by both PC and PH.”—Medical student 1
		“The community immersions definitely prepared me for my professional role as an MHO [municipal health officer] and now working for the Department of Health. It is during the immersions where I saw and experienced the healthcare system.”—Alumnus 2
	Value formation enabling integrated PC and PH	Values (3)
		“Values are developed, such as empathy and health equity.”—Faculty 3
		“The community exposures developed in us values that enable us to integrate PC and PH. [Values] such as compassion drives us not just to provide treatment but also to go the extra mile to conduct health teaching to help control and maintain their health status.”—Medical student 8
		“We [medical students] have experienced how to efficiently deliver services to the community despite resource constraints.”—Medical student 1
Healthcare system outcomes	Improved delivery of health services	Delivery of service (6)
		“Our relationship with the rural health unit has improved, since the students have expanded some of the services to our *barangay*.”—Community stakeholder 7
		“We [medical students] were able to show the communities that health and health-related services are available for them and that they can also lobby for access to services.”—Medical student 5
	Improved continuity of care	Continuity of care (5)
		“In trying to target hypertension in the community, we identified individuals with hypertension then we were able to provide PC services by referring them to the PC centers for access to medicine. We were able to bridge these services to the community members.”—Medical student 1
		“Medical students partner with local district hospitals. As a result, community members are able to access treatment. At the same time, community members become aware of services available for them in the hospitals.”—Community preceptor 1
	Availability of human resources for health	Physicians in the community (3)
		“Effects on students include their choice of practice location, leaning towards rural areas that increased health human resource. [ADZU-SOM] provided doctors in previously doctor-less areas. This is possible because the community immersions developed the desire in them to address population healthcare needs.”—Faculty 2

^a^ Edited for brevity, clarity, and correctness. PC, primary care; PH, public health.

## Discussion

This study explored how primary care and public health were integrated in medical students’ training in Zamboanga Peninsula, Philippines. We analyzed how a curriculum based on social accountability and CEME for training future physicians achieved the integration of primary care and public health. Medical students initiated activities in communities that provided platforms for integration in practice. Public health activities carried out in Mahayag and Tampilisan represented a diverse range of activities, such as stakeholder engagement, policy development, system enhancement, infrastructure and capacity building, resource allocation, health education, and advocacy, which might give an impression there were more public health activities than primary care activities performed. However, primary care activities, such as blood pressure screening and diagnoses were, in practice, performed repeatedly as part of continuing monitoring and evaluation of the health of individual patients. Here we argue that assessing the integration of primary care and public health should not be viewed in an additive way based on the number of activities carried out for each function but by the mutually enhancing contribution of each function when practitioners perform both in the community setting.

The curricular components of outcome-based medical education allowed students to focus on achieving competencies required for earning a dual MD-MPH degree emphasizing skills, practical knowledge, and competencies [39]. Graduates of the ADZU-SOM medical curriculum are envisioned to be physicians who are not only clinicians—they are also leaders and managers, teachers and learners, and researchers. They are envisioned to be competent not only in biomedical knowledge and technical skills but also in the “soft” social and political skills that are important for health managers in local levels in the Philippines [40] and which enable them to be agents of change as they manage the health of individual patients and simultaneously improve population health in the community. The teaching-learning methods, including the community immersions and hospital rotations, provided medical students with opportunities to engage in communities without sacrificing training in the hospital setting. Problem-based—rather than discipline-based—learning modules ensured primary care and public health concepts were taught in complementarity, not in isolation.

Primary care and public health integration was enabled by factors consistent with previous studies [31]. Government support for the initiative and alignment of institutional goals within the Zamboanga Peninsula context are systemic factors for enabling integration. For example, the priorities of the Department of Health served as core curricular content [18], which led to a shared agenda between ADZU-SOM and its partner communities and government agencies. Organizational factors include committed and competent faculty members for integrating primary care and public health, as well as allocating sufficient time for integration in order for outcomes to materialize [18]. Other facilitating factors include faculty members who were also graduates of the same program and working in the same communities where medical students engaged in, and thus served as role models for the students’ future career choices. A determinant of successful integration was the shared purpose between the school and the communities. The first month of the students’ community engagement was dedicated to establishing rapport and strong ties with communities to identify mutual goals.

Our findings suggest that integrating primary care and public health need not wait for health professionals to be part of the active health workforce for integration to take place. Integration begins even during the early stages of training which influence the career trajectories of graduates who possess the integrative mindset as they later practice as both primary care providers and public health practitioners in the health system. The ability to integrate primary care and public health in practice will help address health inequities as primary care providers treat individual patients with the broader health system in mind, and public health practitioners implement programs and policies with an appreciation of how these might affect individual patients. Integration can serve as a steppingstone toward achieving UHC through an effective, comprehensive primary care approach with a strong, proactive public health function. Physicians educated and informed about the value of integrated primary care and public health would play an essential role in integrated care delivery systems for improving the health of the population.

Our study aligns with previous findings that integration results in a range of positive outcomes evident across groups and systems [38]. Individual and community outcomes included changes in health-seeking behaviors, better health outcomes, and empowered community leaders. For medical students, community experiences facilitated a deeper understanding of the complementary value of primary care and public health in health systems. For instance, integration improved access and continuity of care from the identification of patients with hypertension or malnutrition to their referrals to higher levels of care in the health system. Although demonstrating causality extends beyond the scope of our study—study participants reported perceived improvements in case findings, treatment compliance, and health behaviors in the communities as a result of the engagement. While acknowledging that improvements in population health is multifactorial, since the establishment of ADZU-SOM in Zamboanga Peninsula, poverty incidence in the region declined to 23.4% by 2021 (versus 13.2% national average) [41]. Recent data also indicate that births delivered by non-skilled attendants declined to 22.9% (versus 15.6% national average) in 2017, although only 28.1% of children receive age-appropriate vaccinations by 35 months of age (versus 33.4% national average) [42]. Infant mortality rate increased to 20 per 1,000 live births (versus 21 per 1,000 national average) [42]. These indicators suggest the need to sustain the presence of ADZU-SOM medical students in the rural communities to contribute to the delivery of integrated primary care and public health services that help improve the health indicators in the region more broadly.

Due to the national policy on UHC in the Philippines, the Commission on Higher Education has called for medical schools and other health professional schools to reorient their curriculum towards Primary Healthcare [8]. Training institutions may consider using their curricula as the foundation for integrating primary care and public health with the social accountability and CEME approach of ADZU-SOM serving as a model.

Although primary care and public health practices are interdisciplinary by nature, we focused only on medical students and physicians in this study. Despite comprising a relatively small component of the public health workforce, physicians often serve in leadership roles in health systems [43]; thus, physicians with a deeper understanding of both primary care and public health are needed to strengthen health systems. However, further research should explore the integration of primary care and public health in other equally important health professions, such as nursing, midwifery, and dentistry.

### Conclusion

The divergence of primary care and public health has led to missed opportunities for synergistically addressing individual and population health. The integration of primary care and public health is possible in practice by starting with the training of health professionals such as physicians through a curriculum based on social accountability and CEME. The significant amount of time devoted to community immersions during the training of medical students provided opportunities for practicing integrated primary care and public health that influenced personal orientations and the health situation in the communities. Integration in practice continued after training when alumni found themselves serving as primary care and public health practitioners in the region. The experience of training future physicians in Zamboanga Peninsula in the Philippines offers lessons on how the divergence could be addressed. Through integration during training in communities, medical students serve as primary care providers and public health practitioners to improve both the health of individuals and the health of communities.
