# Supplementary material for: The Integration of Primary Care and Public Health in Medical Students’ Training Based on Social Accountability and Community-Engaged Medical Education
Source: Int J Public Health. 2023 Jan 26;68:1605359. doi: 10.3389/ijph.2023.1605359 (PMC9908606; doi:10.3389/ijph.2023.1605359)
Supplement: Supplementary file 1 [file DataSheet2.pdf]

Supplementary File 2: Community Health Plan (CHP) Activity Checklist

**Barangay, Municipality**		**Year**	
**Name of Activity**			
**Problems Addressed**			
**Type of Activity**		**[ ] Primary care**	**[ ] Public Health**
**Target Audience**		**Frequency**	
**How was it done?**<br>***Identify specific activities/factors that facilitate integration of public health and primary care, for example: Coordinating health care services for individuals, Applying a population perspective to clinical practice, Identifying and addressing community health problems, Strengthening health promotion and disease prevention, Collaborating around policy, training, and research, etc.***			
**Outcomes**<br>***Health indicators, health-seeking behaviors, health knowledge, access to health, economic benefits, public health improvements (e.g. water & sanitation, social connectivity, etc.)***			
**Challenges**<br>***Hindrances to implementation, evaluation, and monitoring***			
