# Supplementary material for: The Integration of Primary Care and Public Health in Medical Students’ Training Based on Social Accountability and Community-Engaged Medical Education
Source: Int J Public Health. 2023 Jan 26;68:1605359. doi: 10.3389/ijph.2023.1605359 (PMC9908606; doi:10.3389/ijph.2023.1605359)
Supplement: Supplementary file 2 [file DataSheet3.pdf]

Supplementary File 3. Interview and FGD Guide

Below are the introductory activities you need to perform at the beginning of each interview:

a. Thank the participant for agreeing to be interviewed.
b. Introduce yourself, the school you are from, and the location of the school.
c. Hand them the informed consent form and feedback sheet. Ask them to complete the consent form and return to you; they may keep the information sheet.
d. Provide the background to the project.
e. Advise them that the interview is confidential, and that you will not use their name while the tape is recording.
f. Turn on the recording device. State the date, time, and location.
d. Introduce the type of questions that will be asked to the participant. Then start the interview with question one.

Questions to be asked to each participant group

COMMUNITY STAKEHOLDERS
A signed information sheet has been returned…………………………………………….
Recording device is turned on…………………………………………………………………
Date, time, and location spoken into recording device……………………………………

Q1. Are you aware of the ADZU-SOM student engagement activities present in your community? Can you describe them? (NOTE: List all activities they are aware of; May focus succeeding questions on activities concerning malnutrition and hypertension.)

Q2. Can you describe the benefit you got from this activity? (NOTE: Ask the benefit for each activity; Identify student-led primary care and public health activities.)

Q3. Since the students have been serving your community, what are the key changes you have seen in the community? (NOTE: Ask positive or negative—social, health, infrastructure, etc. changes.)

Q4. Finally, can you identify/explain what led to that change happening in the community? PROMPT: May ask what factors contributed to the change. (NOTE: Explore/Identify facilitating factors or barriers to integration of primary care and public health in the community.)

MEDICAL STUDENTS

A signed information sheet has been returned……………………………………….

Recording device is turned on…………………………………………………………….

Date, time, and location spoken into recording device…………………………….

Q1. What is your understanding of primary care and public health? Describe their similarities and differences. PROMPT: May provide operational definition of primary care and public health.

Q2. How do you think primary care and public health concepts/activities are integrated in the ADZU-SOM curriculum? (NOTE: Identify specific curricular components/activities that are either primary care, public health, or both.)

Q3. What is your understanding of the primary goal of ADZU-SOM's community engagement?

Q4. Can you recount a particular experience/s as a student in ADZU-SOM where you applied and/or integrated primary care and public health? (NOTE: List all community engagement activities that are primary care, public health, or both.)

Q5. What have those experience/s mentioned above taught you? (NOTE: Identify each experience/community engagement activities and identify primary care and public health competencies acquired.)

Q6. Can you describe particular community engagement activities you implemented to address health needs concerning hypertension and malnutrition? Furthermore, describe their outcomes.

Q7. Finally, what do you think contributed to the community outcomes mentioned above? (NOTE: Explore students' perspectives on facilitating factors and/or barriers to integrating primary care and public health.)

FACULTY MEMBERS

A signed information sheet has been returned…………………………………………….

Recording device is turned on……………………………………………………………………

Date, time, and location spoken into recording device………………………………

Q1. Can you describe the nature of your involvement in ADZU-SOM and the years of service you have rendered?

Q2. Can you describe how primary care and public health concepts/activities are integrated in the ADZU-SOM curriculum? (Identify specific curricular components/activities that are either primary care, public health, or both.)

Q3. What effects do you think the school's curriculum (integrating primary care and public health) has had on graduate's commitment to address population health needs (improved chronic disease management, improved maternal and child health, improved communicable disease control, etc.) (NOTE: May explore the primary care and public health competencies taught to the students.)

Q4. Can you describe the factors or barriers that caused the effect/s you mentioned above? (NOTE: Explore faculty perspective on facilitating factors and/or barriers to integrating primary care and public health.)

Q5. In your experience, have students and graduates of the school made any noticeable improvements on the population health needs of the communities in the region? Please describe.

ALUMNI

A signed information sheet has been returned……………………………………………

Recording device is turned on…………………………………………………………………

Date, time, and location spoken into recording device………………………………

Q1. Can you describe the nature of your current work and the years of service you have rendered?

Q2. Did the school's curriculum (integrating primary care and public health) and community engagement experience have any impact on your attitudes to addressing population health needs, community service and/or caring for the underserved? If yes, please give an example. (PROMPT: Has your attitude or values changed since your experience?)

Q3. Can you recall an experience where you integrated primary care and public health concepts as medical students and what outcomes or impacts did it result in? (NOTE: Identify key areas of collaboration to improve integration of primary care and public health.)

Q4. Can you identify/explain what led to the outcomes and impacts mentioned above (Q3)? (NOTE: Explore/Identify facilitating factors or barriers to integration of primary care and public health in the community.)

Q5. Finally, can you describe what you have learned during the experiences mentioned above (Q3) and how your experience in integrating primary care and public health has prepared you to be able to perform the functions required of you in your current position. (NOTE: Identify primary care and public health competencies learned.)

FACULTY COMMUNITY PRECEPTORS

A signed information sheet has been returned…………………………………………

Recording device is turned on……………………………………………………………………

Date, time, and location spoken into recording device……………………………

Q1. Can you describe the nature of your involvement in ADZU-SOM as community preceptor and the years of service you have rendered?

Q2. Can you describe how primary care and public health concepts/activities are integrated in the community engagement activities? (Identify specific curricular components/activities that are either primary care, public health, or both.)

Q3. Since the students have been serving your community, what are the key changes you have seen in the community? PROMPT: May focus on activities addressing Hypertension and Malnutrition (NOTE: Ask positive or negative-social, health, infrastructure, etc. changes.)

From the above question: Regarding the key changes seen in the community, ask:

Q4. Can you identify/explain what led to that change happening in the community? PROMPT: May ask what factors contributed to the change. (NOTE: Explore/Identify facilitating factors or barriers to integration of primary care and public health in the community.)

