# Supplementary material for: The Integration of Primary Care and Public Health in Medical Students’ Training Based on Social Accountability and Community-Engaged Medical Education
Source: Int J Public Health. 2023 Jan 26;68:1605359. doi: 10.3389/ijph.2023.1605359 (PMC9908606; doi:10.3389/ijph.2023.1605359)
Supplement: Supplementary file 3 [file DataSheet1.pdf]

Supplementary File 1

Demographic characteristics of study participants

	In-depth interviews N= 4	*Focus group discussions* N= 40
**Sex**		
Male	2 (50%)	16 (40%)
Female	2 (50%)	24 (60%)
**Age**		
20–30 years old		13 (32%)
31–40 years old	1 (25%)	8 (20%)
41–50 years old	3 (75%)	7 (18%)
51–60 years old		9 (23%)
61–70 years old		1 (2%)
71–80 years old		2 (5%)
**Religion**		
Roman Catholic	3 (75%)	37 (93%)
Islam	1 (25%)	1 (2%)
Protestant		2 (5%)
**Informant category**		
Faculty/community preceptors	2 (50%)	6 (15%)
Medical students		13 (33%)
Community stakeholders		21 (52%)
Program graduates/Alumni	2 (50%)	
